# Society Meetings

**Published:** 1910-02

**Authors:** 


### SOCIETY MEETINGS.

The International American Congress of Medicine and Hygiene in commemoration of the first centenary of the May revolution of 1810, under the patronage of His Excellency, the President of the Argentine Republic, will be held May 25, 1910, in Buenos Aires, Argentine Republic. In order to facilitate the contribution of papers and exhibits from the United States, there has been appointed by the President of the Congress, Dr. Eliseo Cantôn, and the Minister of the Argentine Republic at Washington, a committee of propaganda of which Dr. Charles H. Frazier, Philadelphia, Pa., is chairman and Dr. Alfred Reginald Allen, Philadelphia, Pa., is secretary.

The Congress has been divided into nine sections, each section being represented in the United States by its chairman in this committee of propaganda as follows:

Section 1—Biological and Fundamental Matters, W. H. Howell, Chairman, Baltimore, Md.; Section 2, Medicine and its Clinics, George Dock, Chairman, New Orleans, La,; Section 3, Surgery and its Clinics, John M. T. Finney, Chairman, Baltimore, Md.; Section 4, Public Hygiene, Alexander C. Abbott, Chairman, Philadelphia, Pa.; Section 5, Pharmacy and Chemistry, David L. Edsall, Chairman, Philadelphia, Pa.; Section 6, Sanitary Technology, W. P. Mason, Chairman, Troy, New York; Section 7, Veterinary Police, Samuel H. Gilliland, Chairman, Marietta, Pa.; Section 8, Dental Pathology, George V. I. Brown, Chairman, Milwaukee, Wis.; Section 9, Exhibition of Hygiene, Alexander C. Abbott, Chairman, Philadelphia, Pa.

It will not be necessary for one contributing a paper or exhibit to the congress to be present in person. Arrangements will be made to have contributions suitably presented in the absence of the author. The official languages of the Congress will be Spanish and English.

Members of the following professions are eligible to present papers or exhibits: Medicine, Pharmacy, Chemistry, Dentistry, Veterinary Medicine, Engineering, and Architecture. Papers may be sent direct to the chairman of the particular section for which they are intended, or to Dr. Alfred Reginald Allen, secretary, 111 South 21st street, Philadelphia, Pa.

---

The Buffalo Academy of Medicine held meetings during the month of January, 1910, as follows:

*Section on Surgery.*—Tuesday evening, January 4, 1910.

Program: Report of a New Functional Test of Renal

Efficiency in Surgical Diseases of the Kidney, John Geraghty, of Baltimore, Chief of the Genitourinary Clinic at Johns Hopkins Hospital; Differential Diagnosis of Prostatic Disorders, with Special Reference to Cancer, Henry Adsit. The discussion was opened by J. A. Gardner.

*Section on Medicine.*—Tuesday evening, January 11. Program: The Use of Sea Water in the Treatment of Disease, Burt J. Maycock; Determinable and Indeterminable Factors in the Etiology of Typhoid Fever, L. L. Lumsden, United States Public Health and Marine-Hospital Service, Washington, D. C.

*Section on Obstetrics and Gynecology.*—Tuesday evening, January 18. Program: The Influence of Temperament on Pelvic Diseases, M. D. Mann; Borderland between Obstetrics and Gynecology, P. W. Van Peyma. Discussion opened by C. C. Frederick and L. Schroeter.

*Section on Pathology.*—Tuesday evening, January 25. Program: The Toxin Treatment of Sarcoma, William B. Coley of New York.

---

The Rochester Academy of Medicine held meetings during the month of January, 1910, at the Hotel Seneca, as follows:

*General Medicine.*—Wednesday, January 5, 1910. Program: Nature of Hypnotic Phenomena, Haydon Rochester.

*Obstetrics and Gynecology.*—Wednesday, January 19. Program: A Spontaneous Tearing of the Cervix Posterior during Abortion, Lacey B. Darling, Palmyra, N. Y. Discussion opened by S. L. Elsner.

*Public Health.*—Wednesday, January 26. Program: The Function of County Laboratories in the Preservation of the Public Health of Country Communities, C. W. Hennington, (by invitation). Discussion opened by O. J. Hallenbeck, Canandaigua.

---

The Southern Surgical and Gynecological Association held its most recent annual meeting at Hot Springs, Va., electing the following-named officers: president, W. O. Roberts, of Louisville; vice-presidents, J. C. Bloodgood, of Baltimore and Lewis C. Morris, of Birmingham; treasurer, W. S. Goldsmith, of Atlanta; and secretary W. D. Haggard, of Nashville. The next meeting will be held at Nashville, in December, 1910.

